# CDK2 inhibition disorders centrosome stoichiometry and alters cellular outcomes in aneuploid cancer cells

**DOI:** 10.1080/15384047.2023.2279241

**Published:** 2023-11-30

**Authors:** Zibo Chen, Xi Liu, Masanori Kawakami, Xiuxia Liu, Allison Baker, Aayush Bhatawadekar, Liliya Tyutyunyk-Massey, Kedar Narayan, Ethan Dmitrovsky

**Affiliations:** a Molecular Pharmacology Program, Frederick National Laboratory for Cancer Research, Frederick, MD, USA; b Center for Molecular Microscopy, Center for Cancer Research, National Cancer Institute, National Institutes of Health, Bethesda and Cancer Research Technology Program, Frederick National Laboratory for Cancer Research, Frederick, MD, USA

**Keywords:** Centrosome stoichiometry, CDK2 inhibition, cancer aneuploidy, lung cancer, anaphase catastrophe

## Abstract

Cyclin-dependent Kinase 2 (CDK2) inhibition prevents supernumerary centrosome clustering. This causes multipolarity, anaphase catastrophe and apoptotic death of aneuploid cancers. This study elucidated how CDK2 antagonism affected centrosome stoichiometry. Focused ion beam scanning electron microscopy (FIB-SEM) and immunofluorescent imaging were used. Studies interrogated multipolar mitosis after pharmacologic or genetic repression of CDK2. CDK2/9 antagonism with CYC065 (Fadraciclib)-treatment disordered centrosome stoichiometry in aneuploid cancer cells, preventing centrosome clustering. This caused ring-like chromosomes or multipolar cancer cells to form before onset of cell death. Intriguingly, CDK2 inhibition caused a statistically significant increase in single centrioles rather than intact centrosomes with two centrioles in cancer cells having chromosome rings or multipolarity. Statistically significant alterations in centrosome stoichiometry were undetected in other mitotic cancer cells. To confirm this pharmacodynamic effect, CDK2 but not CDK9 siRNA-mediated knockdown augmented cancer cells with chromosome ring or multipolarity formation. Notably, engineered gain of CDK2, but not CDK9 expression, reversed emergence of cancer cells with chromosome rings or multipolarity, despite CYC065-treatment. In marked contrast, CDK2 inhibition of primary human alveolar epithelial cells did not confer statistically significant increases of cells with ring-like chromosomes or multipolarity. Hence, CDK2 antagonism caused differential effects in malignant versus normal alveolar epithelial cells. Translational relevance was confirmed by CYC065-treatment of syngeneic lung cancers in mice. Mitotic figures in tumors exhibited chromosome rings or multipolarity. Thus, CDK2 inhibition preferentially disorders centrosome stoichiometry in cancer cells. Engaging this disruption is a strategy to explore against aneuploid cancers in future clinical trials.

## Introduction

Genomic instability is a cancer hallmark.^1^ Aberrant centrosome numbers frequently occur in cancer cells^2^ and are associated with genomic instability.^3^ Mitosis with supernumerary centrosomes leads to abnormal chromosome segregation and aneuploidy in the cellular progeny.^4,5^ While many cancer cells with abnormal chromosomes are eliminated through apoptosis, some gain a growth advantage and acquire resistance to chemotherapy or targeted cancer therapy.^3,6–8^ Such aneuploidy is associated with an aggressive tumor biology and unfavorable clinical outcome.^9^

The centrosome is composed of diverse proteins with two centrioles surrounded by pericentriolar material (PCM) that serves as the microtubule organizing center.^10^ Centrosomes control polarity as well as specific interphase and mitotic microtubule-dependent processes including chromosomal segregation during mitosis.^10^ To maintain chromosome stability, both cell cycle progression and the centrosome duplication cycle must be tightly coordinated.^11^ In different cancers, deregulation of the centrosome cycle causes numerical and structural centrosome abnormalities.^4^ Elucidating the precise role played by the centrosome cycle in cancer biology is under active pursuit.^4,12^ This has been a subject of study since the seminal work of Hansemann and Boveri over a century ago that identified chromosomal abnormalities, centrosome alterations and aneuploidy as hallmarks of cancer, as reviewed.^4^

Cell cycle machinery regulators also play a critical role in the centrosome cycle,^11,13,14^ including cyclin-dependent kinase 2 (CDK2), as explored here. Inhibition of cyclin A/E binding to the centrosome complex antagonizes CDK2-dependent S phase entry.^15^ CDK2 has a key role in centrosome duplication. This involves interactions between CDK2 and the SKP1-Cullin-Fbox E3 (SCF) ligase β-transducing-repeat-containing protein (βTrCP) that affects centriole biogenesis by suppressing βTrCP dependent STIL.^16^ CDK2 causes Mps1 destabilization within centrosomes to promote centriole duplication.^17^ Centrosomal localization of cyclin E-CDK2 is required for initiation of DNA synthesis.^18^

The centrosome duplication cycle begins at the G1-S phase when the pair of centrioles dissociates.^5,13–19^ Centrosome disengagement in late G1 is licensed by the phosphorylation of nucleophosmin (NPM) via cyclin E/CDK2 complexes.^13,20–25^ Prior work revealed that centriole disengagement occurs during anaphase when CDK2 is needed for centriole duplication in the next cell cycle.^25^ CDK2 is dispensable for normal centrosome duplication, but required for oncogene-induced centrosome overduplication.^26^ Mechanistic studies conducted in CDK4^−/−^ mouse embryonic fibroblasts (MEFs) established that centrosomes fail to separate at G1/S phase, while centrosomes achieve premature separation in CDK2^−/−^ MEFs.^27^ Activation of E2F transcription factors and functional CDK2-cyclin A complex are collectively required for centriole duplication.^25^ Inactivation of E2F3 can prevent premature centriole duplication, regulate centrosome amplification, and maintain chromosome stability by controlling cyclin E levels and cyclin E-dependent kinase activity.^28^

The role of abnormal centrosome stoichiometry (typically centrosome amplification) in cancers is cancer-context dependent.^9,29^ For example, centrosome amplification is associated with the clinical biology of hormone receptor-negative primary invasive breast cancers^30^ as well as in advanced stages of triple-negative breast cancer, a clinically-aggressive malignancy.^31^ In addition, a centrosome amplification mouse model having truncated alleles of the adenomatous polyposis coli (APC^Min^) tumor suppressor and doxycycline-inducible Polo-like Kinase 4 (PLK4) (APC^Min/+^; Plk4^Dox^) exhibited higher intestinal tumor incidence as compared to APC^Min^ mice.^32^ Also, increased centrosome amplification can promote breast cancer migration and invasion as well as alter clinical outcomes.^33,34^ Thus, centrosome alterations affect cancer biology.

Pharmacological approaches exist to target supernumerary centrosomes and to affect chromosome instability in cancer cells.^35,36^ These include the drug Palbociclib that inhibits CDK4/6. Palbociclib was FDA-approved for a subset of breast cancers.^37,38^ Notably, the PLK4 inhibitor CFI-400945 augmented supernumerary centrosomes and suppressed lung cancer cell proliferation by promoting polyploidy and apoptotic death.^39^ The Aurora kinase A inhibitor Alisertib (MLN8237)^40,41^ is under clinical trial investigations in oncology, with varying efficacy. This underscores the need to find ways to enhance the clinical activity of this and related agents. In this regard, the Tyrosine Threonine Kinase (TTK) inhibitor CFI-402257 triggered aberrant chromosomal segregation and extensive aneuploidy in lung cancers and other cancers.^42,43^

Our prior work found that genetic or pharmacologic inhibition of CDK2 antagonized centrosome clustering and caused cancer cells with supernumerary centrosomes to undergo multipolar mitosis and chromosome mis-segregation with the consequence of apoptotic death of the progeny, termed anaphase catastrophe.^44–49^ Enhanced anaphase catastrophe prevented preexisting supernumerary centrosomes from clustering and eradicated aneuploid cancer cells while relatively sparing immortalized bronchial epithelial cells.^44–49^ The pan-CDK or selective CDK2 inhibitors seliciclib,^44^ dinaciclib,^50^ CCT68127^49^ and CYC065 (Fadraciclib)^48^ each caused anaphase catastrophe. Notably, CCT68127^49^ and CYC065^48^ that each inhibit CDK2 activity also conferred statistically-significant repression of tumor growth in mice bearing syngeneic or patient-derived xenografts (PDXs) of lung cancers. CYC065-treatment of these mice led to lung cancers having multipolar or abnormal mitosis.^48^ Studies reported here elucidate pathways that are present in *in vivo* cancers after CDK2 antagonism. Several of these and other cell cycle targeting agents are in clinical trial testing, attesting to the translational relevance of these agents.^35^

This study explores the basis for induced anaphase catastrophe. Evidence provided here implicates the disruption of centrosome stoichiometry after CDK2 antagonism as associated with the death of aneuploid cancer cells while relatively sparing normal human alveolar epithelial cells. Prior work found that chromosome rings are transient intermediates that follow chromosome segregation in metaphase during normal cell mitosis.^51^ The fates of aneuploid cancer cells that exhibit chromosome rings after CDK2 inhibition were previously unknown. Likewise, the precise relationship between CDK2 inhibition and the appearance of either multipolar mitosis or chromosomal rings was not determined.

This study addresses these prior knowledge gaps. To further elucidate the nature of these chromosomal structures relative to potential centrosome abnormalities, centrosomal immunofluorescent staining and focused ion beam scanning electron microscopy (FIB-SEM)^52,53^ were employed after pharmacologic (CYC065, a CDK2/9 inhibitor) or genetic (siRNA)-mediated repression of CDK2. Rescue experiments confirmed the critical role played by CDK2 in conferring this death program. Indeed, engineered CDK2, but not CDK9 over-expression, reversed the presence of cancer cells having multipolar mitoses or chromosome ring formations after treatment with CYC065 or transfection of siRNAs that targeted CDK2. Intriguingly, differential effects were seen after CYC065 treatment of lung and other cancers versus primary human alveolar epithelial cells. The translational relevance of these studies was confirmed by examination of CYC065-treated syngeneic lung cancers in mice. Thus, this report reveals a pharmacologically-tractable way to disrupt centrosomal stoichiometry and trigger death of aneuploid cancer cells. Together, these findings inform ways to combat aneuploid tumors like lung cancers.

## Results

### Chromosome rings and multipolar mitoses after CDK2/9 inhibition

**Figure 1.**
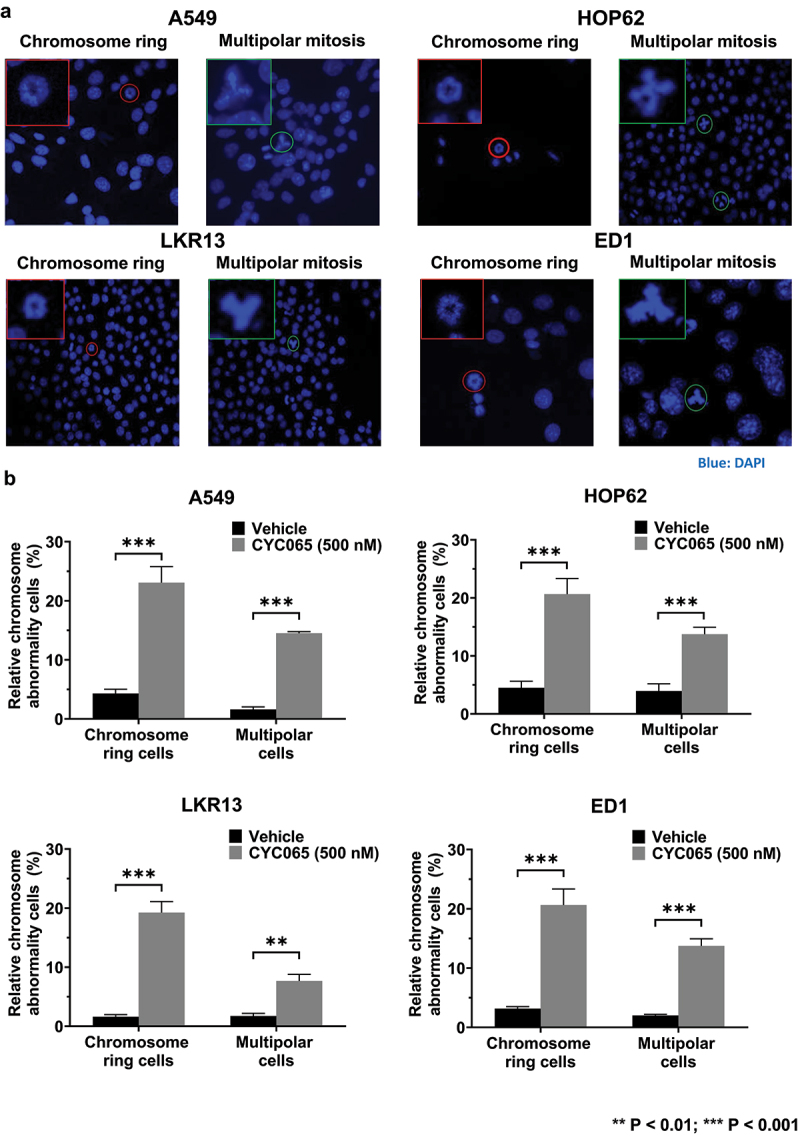
Chromosome rings and multipolar mitoses were respectively increased in lung cancer cells by CYC065-treatment. (a) Human (A549 and HOP62) and murine (LKR13 and ED1) lung cancer cell lines were independently treated with CYC065 (500 nM) for 24 hours. Immunofluorescent assays elucidated the presence of chromosome rings or multipolar mitotic cells in each of these examined cancer cell lines. Blue signals indicated DAPI staining of DNA. In each image, the red or green circles highlighted the cell of interest. The red or green box showed the higher magnification of the cancer cell of interest. (b) CYC065-treatment increased the proportion of cells having chromosome rings or multipolar mitoses in human (A549 and HOP62) as well as murine (LKR13 and ED1) lung cancer cells as compared to vehicle controls as quantified by immunofluorescent assays. Error bars represented standard deviations with the symbols indicating **P < .01 and ***P < .001, respectively.

CDK2/9 inhibitor (CYC065)-treatment caused chromosome rings or multipolar mitoses to form in human (A549 and HOP62) and murine (LKR13 and ED1) lung cancer cells (Figure 1a). Notably, the presence of chromosome rings was found in subsets of these treated cancer cells (Figure 1a). Statistically-significant increases in both chromosome rings and multipolar mitoses were detected in human (A549 and HOP62) and murine (LKR13 and ED1) lung cancer cell lines following CYC065 (500 nM)-treatment as compared to vehicle controls (Figure 1b). These results indicate that this CDK2/9 inhibitor markedly augmented the percentage of cancer cells with chromosome rings or multipolarity in these examined cancer cell lines.

### CYC065 treatment and chromosome rings

**Figure 2.**
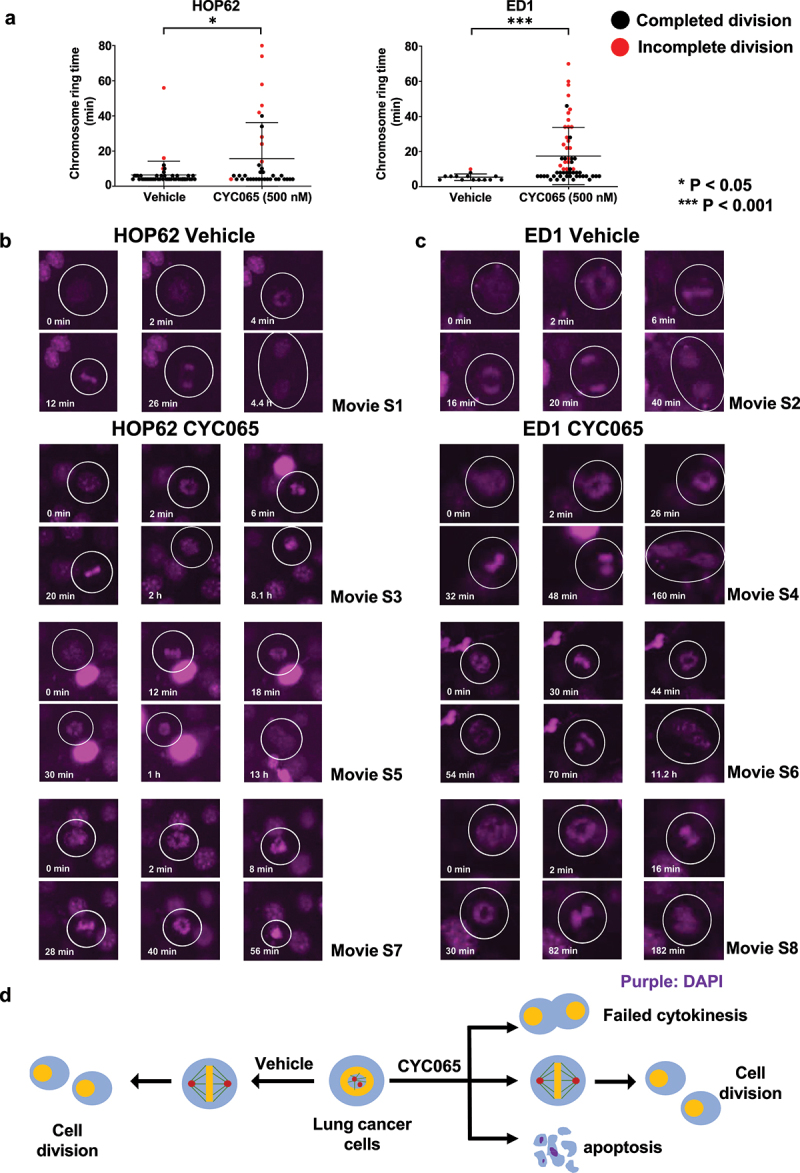
CYC065-treatment produced chromosome rings in HOP62 and ED1 lung cancer cells. (a) CYC065-treatment increased the duration of chromosome rings in HOP62 and ED1 lung cancer cells as compared to vehicle controls as measured by live cell imaging assays for 48 hours (with 2-minute image time intervals). The symbols referred to *P < .05 and ***P < .001, respectively. Representative results are displayed for CYC065 (500 nM)-treatment versus vehicle controls for: (b) HOP62 and (c) ED1 lung cancer cells that respectively exhibited failed cytokinesis in cells having chromosome rings. Purple signals represented NucSpot live 650 nuclear dye staining for DNA. White circles indicated the cell of interest. (d) This diagram summarized the temporal sequence of chromosome ring and bipolar mitosis in representative cancer cells.

Prior work found that chromosome rings are transient intermediates that follow chromosome segregation during bipolar metaphase.^51^ To explore whether CYC065-treatment substantially increased the proportion of cancer cells having chromosome rings, live cell imaging assays were performed in human (HOP62) and murine (ED1) lung cancer cell lines following CYC065 (500 nM)-treatment versus vehicle controls over sequential 2-minute time intervals of study. Findings revealed that the duration of the presence of chromosome rings was statistically-significantly increased after CYC065-treatment of these cancer cells as compared to vehicle controls (Figure 2a).

Intriguingly, a statistically-significant and high proportion of cells having chromosome rings failed to complete mitosis after CYC065 (500 nM)-treatments of HOP62 and ED1 lung cancer cells unlike the minor presence of these intermediates in vehicle controls, as detected in Figure 2a. To investigate the fates of lung cancer cells with chromosome rings, live cell imaging assays were performed in HOP62 and ED1 lung cancer cells after CYC065-treatment versus vehicle controls, with results displayed over 72 hours (2-minute time intervals). While most cancer cells having chromosome rings completed cytokinesis in the vehicle group (Figure 2b,c; movies S1 and S2), a statistically-significantly higher proportion of cancer cells with chromosome rings in the CYC065-treated group exhibited failed cytokinesis or cell death (Figure 2b,c; movies S3-S8). The fates of cells with chromosome rings treated with vehicle or CYC065 are summarized in Figure 2d. CYC065-treatment extended the duration of detection of chromosome rings and caused abnormal mitoses, including failed cytokinesis.

### Appearance of chromosome rings and bipolar metaphases after CDK2 inhibition

**Figure 3.**
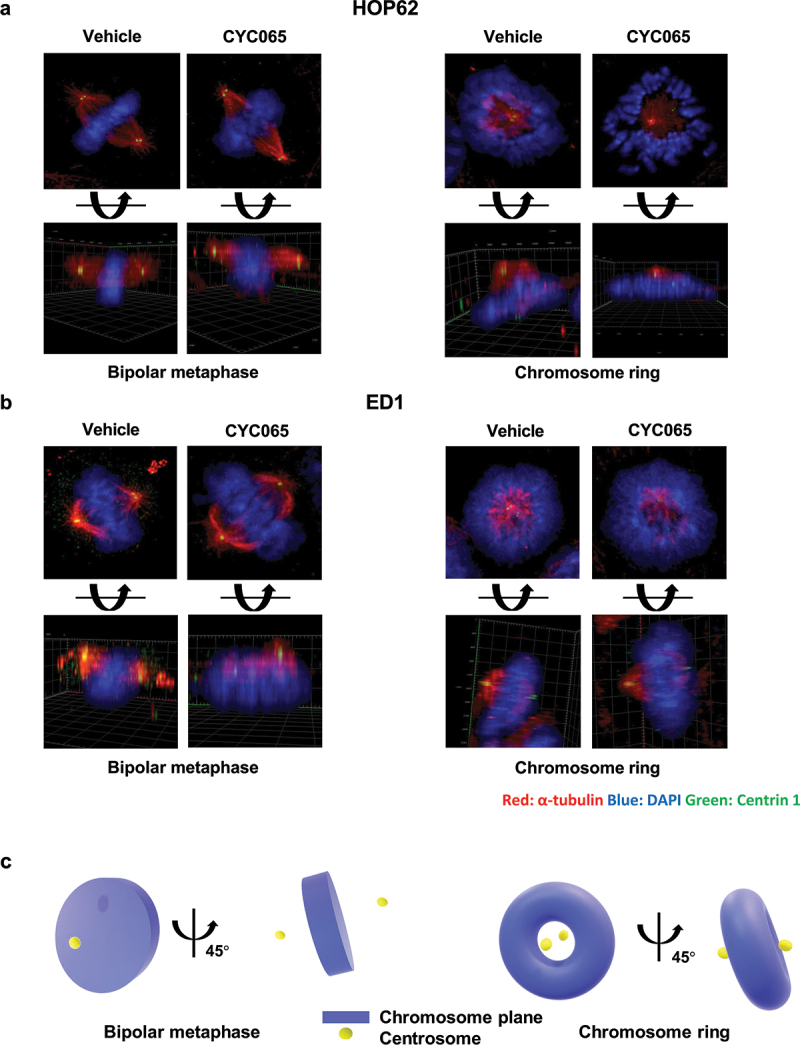
Immunofluorescent assays were independently performed to identify potential centriolar structural alterations in: (a) human HOP62 and (b) murine ED1 lung cancer cells. The red signals indicated α-tubulin staining; blue signals revealed DAPI staining and the green showed Centrin 1 staining. Representative images are shown. (c) The diagram presents the spatial arrangement of centrioles relative to the chromosome planes with a representative chromosome ring or bipolar mitosis depicted.

To independently establish that chromosome rings constitute distinct structures and not simply reflect the angle of imaging of examined metaphase cancer cells, immunofluorescent assays and confocal imaging were each performed in HOP62 and ED1 lung cancer cells treated with CYC065 (500 nM) versus vehicle controls. Immunostaining for α-tubulin and Centrin 1 as well as DAPI staining were done. Confocal microscopy and separately Z-stack imaging revealed after CYC065 inhibition the relative relationship between the displayed centrosomes and the chromosome rings, as compared to cancer cells having bipolar metaphases (see Figure 3a,b and S1). The schematic summary of spatial relationships between the centrosomes and chromosomal planes of cancer cells with bipolar metaphase or chromosome rings are displayed in Figure 3c. The detailed quantification of abnormal centriolar stoichiometry after CYC065 versus vehicle treatment of human and murine lung cancer cells is shown in Figure S2.

Fluorescent imaging of these CYC065-treated cancer cells revealed that chromosome ring structures were prominently increased by CYC065-treatment. A higher resolution of ultrastructural imaging was required to better define these structural changes. Fluorescence microscopy was used to identify specific cells for FIB-SEM analysis followed by imaging and reconstruction of their respective structures using Z sections (see movies S9 and S10).

**Figure 4.**
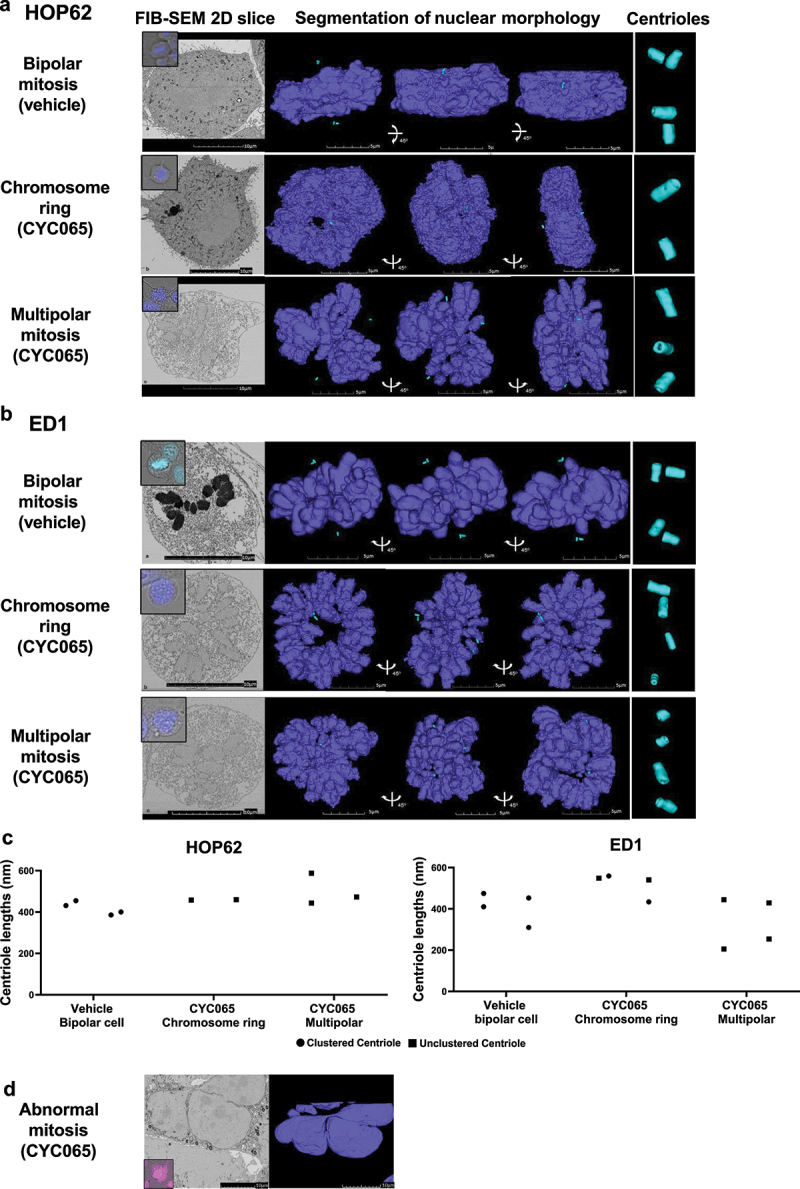
FIB-SEM reconstructions of nuclear morphology and centriolar structures are shown for: (a) human HOP62 and (b) murine ED1 lung cancer cells. FIB-SEM two-dimensional representative slice images, segmentation of nuclear morphology with 0-, 45- and 90-degree angles presented, respectively, and centriole three dimensional images are displayed. (c) Unlike the normal lengths of coupled centrioles in these images of some of the cells in panels a and b, cells with single centrioles exhibited centrioles with altered lengths, as shown in this panel (on left is human HOP62 and on right murine ED1 lung cancer cells). (d) a representative CYC065-treated HOP62 lung cancer cell exhibited abnormal nuclear morphology by FIB-SEM measurements. Purple signals indicated NucSpot live 650 DNA dye staining and teal images displayed condensed chromosomes and centrioles, respectively.

Segmentation of nuclei and centrioles in HOP62 and in ED1 lung cancer cells treated with CYC065 relative to vehicle as a control treatment revealed three predominant structural intermediates. Vehicle-treated cells exhibited condensed chromosomes in a canonical metaphase plate with the corresponding pairs of centrosomes on either side along the central axis. Each centrosome was confirmed to contain two centrioles each, as a bipolar mitotic event (Figure 4a, middle and lower panels, and 4b, middle and lower panels). In marked contrast, CYC056 (500 nM) independently treated HOP62 and ED1 lung cancer cells prominently had chromosome ring or multipolar structures with centriole abnormalities, as in Figure 4a,b. The displayed reconstructions allow for the observed volumes to be rotated by use of Z-stack analyses. These Z-stack images shown in the lower panels of Figure 3a,b, respectively, confirmed that these were unique conformations and not merely different views taken of similar structures. Some cells had poorly preserved structures, possibly due to drug exposure or phototoxicity from prolonged fluorescence microscopy exposure times.

Interestingly, there was altered centriole stoichiometry and clustering observed after CYC065-treatment. The displayed HOP62 cancer cell line having a chromosome ring had only one centriole per centrosome. This organelle was located along the central axis of the ring, but these centrioles were separated from each other and on opposite sides of the metaphase plate (Figure 4a). A representative multipolar HOP62 cancer cell also deviated from the expected stoichiometry, with the three detected centrosomes only containing one centriole each (Figure 4a). Surprisingly, these CYC065-treated cells were far advanced in the M phase of the cell cycle without having undergone centriole duplication or alternatively the destruction of two centrioles after duplication. This points out the capacity of CYC065 to decouple or alter the process of centriole duplication from that of the expected progression of the cell cycle.

The examined ED1 murine lung cancer cells having chromosome rings or multipolar mitosis also showed abnormal centriole stoichiometry and spatial arrangements. The two centrioles for a centrosome were spatially separated (Figure 4b, chromosome ring and multipolar mitosis), indicating the suppression of centriole clustering by CYC065-treatment, as previously reported.^49^ Together, these FIB-SEM analyses showed that CYC065-treatement caused two distinct nuclear structural intermediates with abnormal stoichiometry and spatial clustering of the centrioles. Unlike the normal lengths of coupled centrioles in these displayed images those cells shown in Figure 4a,b with single centrioles detected exhibited centrioles with altered lengths, as shown in the panels displayed in Figure 4c.

To better understand the fates of the cells with chromosome rings after CYC065 treatment, cells of interest were stained with NucSpot Live 650 cell DNA dye and monitored under live cell microscopy. Following the appearance of chromosome rings, cell nuclei showed signs of coalescing and appeared to form multinucleated cells after 36 hours of CYC065 (500 nM) treatment. Altered centriole lengths were observed in some CYC065-treated HOP62 human lung cancer cells and in ED1 murine lung cancer cells, as in Figure 4c. FIB-SEM analysis was performed on these cells and revealed that the nuclear membranes formed around the DNA with incompletely separated nuclei, although the chromosomes had decondensed into an interphase-like state (Figure 4d). Given the limitation of imaging these contiguous polynuclear morphologies under conventional light microscopy, further resolution and study of mitotic cells were needed. This was performed at higher resolutions and in three dimensions.

### CYC065 effects on centriole stoichiometry

**Figure 5.**
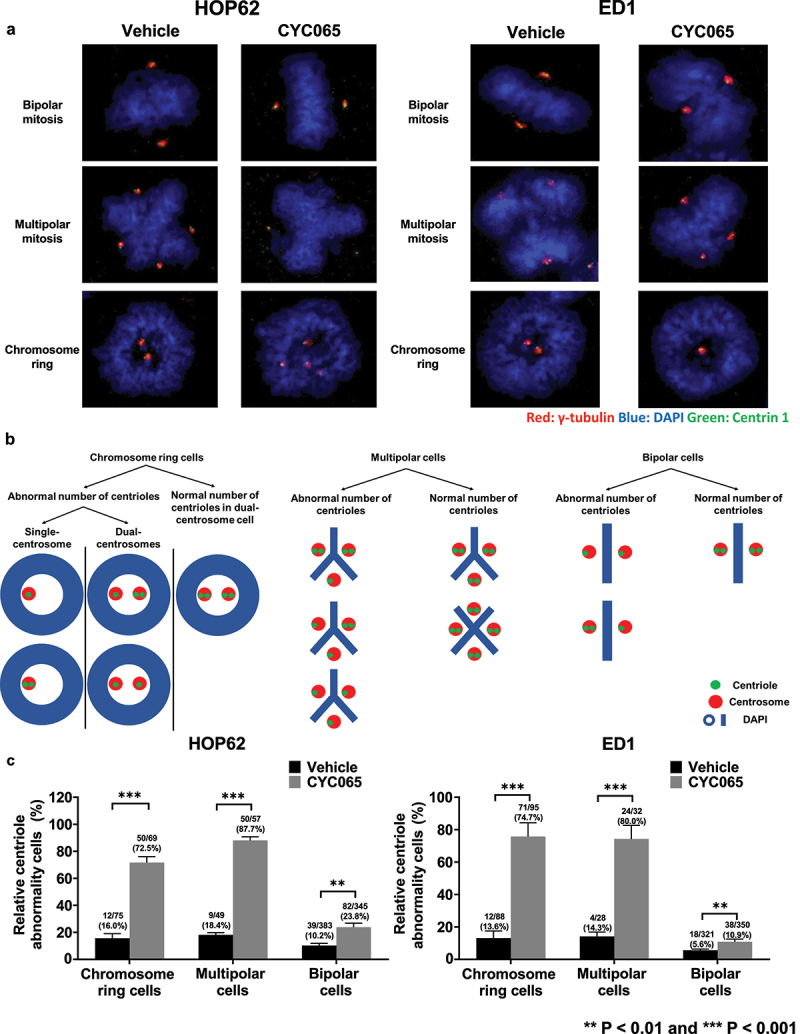
CYC065 treatment effects on centriolar stoichiometry were independently determined in HOP62 and ED1 lung cancer cells. In panel a, HOP62 and ED1 cells were each treated with CYC065 (500 nM), and immunofluorescent staining was independently examined for γ-tubulin (red), DAPI (blue) and Centrin 1 (green) staining, respectively. Representative images are shown for lung cancer cells with bipolar or multipolar mitoses as well for those cells having chromosome rings in each treatment group. Panel b summarized the respective centriolar abnormalities in the indicated lung cancer cells having bipolar, multipolar mitoses or chromosome rings. In panel c, CYC065-treatment increased the percentage of cells with centriolar abnormalities in lung cancer cells having chromosome rings or bipolar mitoses or multipolar mitoses by use of immunofluorescence assays. Error bars are displayed as standard deviations with statistical significance indicated by these symbols: **P < .01 and ***P < .001, respectively.

To confirm and extend the findings obtained from FIB-SEM studies, how CYC065-treatment affected the stoichiometry of centrosome and centriole numbers was assessed by immunofluorescent assays. HOP62 and ED1 lung cancer cells were each treated with CYC065 or vehicle as a control and then immunostained independently for γ-tubulin and Centrin 1 along with DAPI staining for centriole stoichiometry scoring (Figure 5a). Centrosomes with two centrioles were scored as exhibiting a normal centriole stoichiometry, as in Figure 5b. CYC065-treatment statistically-significantly increased the proportion of cells with abnormal centriole stoichiometry as compared to vehicle treatment (Figure 5c). Notably, the ratios of abnormal centriole stoichiometry were increased substantially more in cancer cells with chromosome rings or multipolar mitosis as compared to those cells with bipolar mitoses.

### CYC065 treatment effects in primary human alveolar epithelial and cancer cells

**Figure 6.**
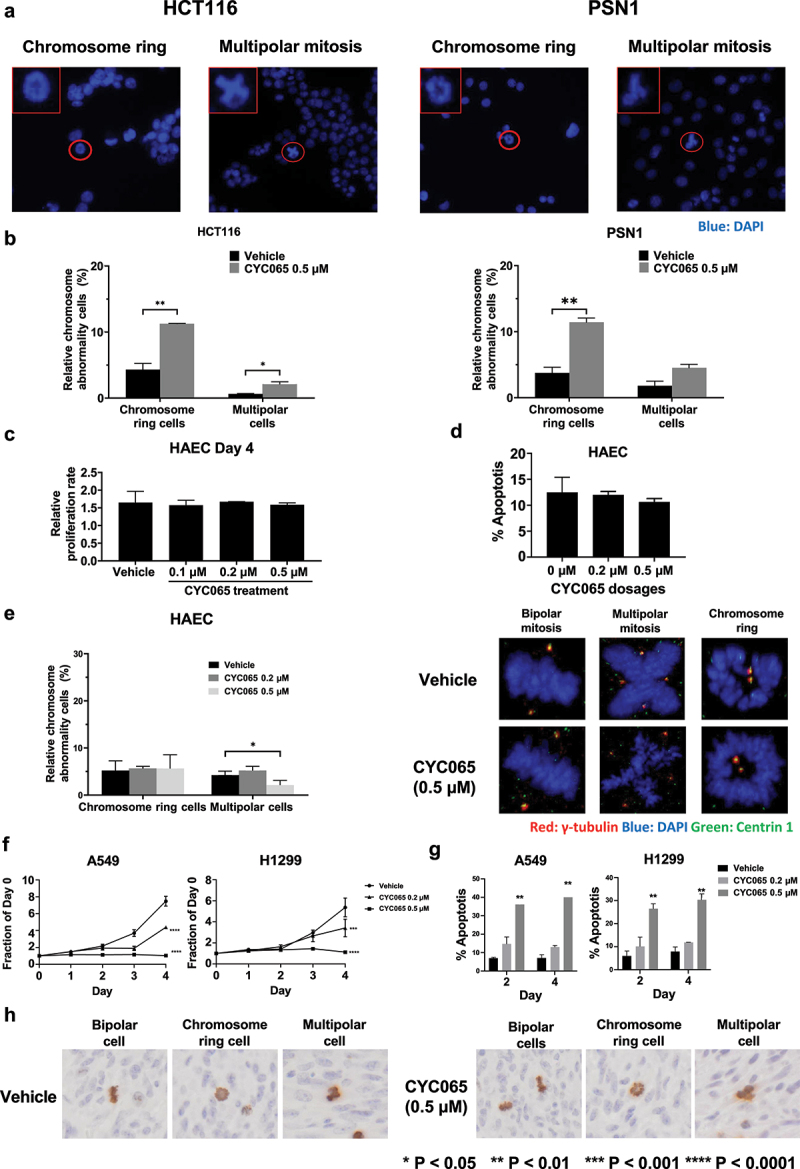
Presence of centrosome rings and multipolar mitoses following CYC065 versus vehicle treatments of colon and pancreatic cancer cells. CYC065 treatment effects are compared to those found in primary human alveolar epithelial cells (HAEC). (a) Both chromosome rings and multipolar mitoses were found in colon (HCT116) and pancreatic (PSN1) cancer cells by use of immunofluorescent assays following treatment with CYC065 (500 nM) as compared to vehicle controls. The red circle indicates the cell of interest; the red box shows the higher magnification of the imaged cell. (b) Quantification of chromosome rings and multipolar mitoses were detected in HCT116 and PSN1 cancer cells following CYC065 versus vehicle control treatment with the symbols *P < .05 and **P < .01, respectively. Error bars are standard deviations. (c) CYC065 treatment does not appreciably affect proliferation of HAEC. Error bars are standard deviations. (d) CYC065 treatment does not elicit statistically significant apoptotic effects in HAEC. Error bars are standard deviations. (e) CYC065-treatment versus vehicle controls and effects in HAEC. In the left panel is shown the percentage of these cells with centrosome abnormalities. Representative bipolar cells as well as those showing either a chromosome ring or multipolar division appear in the right panel. Centrosome abnormalities appear only after CYC065-treatment of mitotic HAEC with chromosome ring or multipolar divisions. Staining indicates: γ-tubulin (red), DAPI (blue), and Centrin 1 (green). (f) In contrast to the CYC065-treatment effects on proliferation and apoptosis of primary HAEC, A549 and H1299 lung cancer cells independently exhibited statistically significant and CYC065 dose-dependent declines in growth and an increase in apoptosis in these cells, as displayed in panel g. Error bars represented standard deviations with the symbols indicating *P < .05; **P < .01; ***P < .001, and ****P < .0001, respectively. (h) Representative images are shown for phospho-histone H3 immunostaining of lung cancers following 4 weeks of CYC065 treatment (versus vehicle controls). As expected from *in vitro* studies, mitotic cancer cells in lung cancers exhibited bipolar, centrosome ring, or multipolar morphologies.

To assess whether chromosome rings or multipolar mitotic cancer cells were detected in other cancer types, colon (HCT116) and pancreatic (PSN1) cancer cell lines were each treated with CYC065 (500 nM); chromosome rings and multipolar cancer cells were each detected in Figure 6a,b. Quantification of these mitotic cancer cells that exhibited either chromosome rings or multipolar cancer cells appear in Figure 6b. These cancer cells when treated with CYC065 (500 nM) increased the proportion of both mitotic figures as compared to controls.

In marked contrast, independent CYC065 treatments at the 200 nM and 500 nM dosages versus vehicle control treatment of primary human alveolar epithelial cells did not reveal an appreciable growth inhibition or increase in apoptosis of these cells, as in Figure 6c,d. The quantification of centrosome staining of mitotic human alveolar epithelial cells treated for 2 days with vehicle or with CYC065 (500 nM) appear in Figure 6e (left panel). Representative images of normal-appearing centrosomes during bipolar divisions of CYC065-treated versus vehicle-treated primary human alveolar epithelial cells are displayed in Figure 6e (right panel) and compared to abnormal centrosomes found in mitotic cells having chromosomal rings or multipolar divisions. As shown in Figure 6e (right panel), abnormal centrosome structures appear in CYC065-treated human alveolar epithelial cells in representative mitotic figures having a chromosomal ring or multipolar division.

As expected from our prior work^35,44–50^ two different lung cancer cell lines, A549 and H1299 had statistically-significant growth inhibitory and apoptotic effects after CYC065 treatments in Figure 6f,g, as compared to vehicle controls. Unlike these examined lung cancer cells, primary human alveolar epithelial cells did not exhibit after CYC065 treatment an increase in mitotic cells exhibiting either centrosome rings or multipolarity in Figure 6e (left panel). Indeed, most mitotic figures had bipolar divisions. To determine if mitotic cancer cells present in lung cancers exhibited bipolar divisions, centrosome rings or multipolar mitosis CYC065 versus vehicle control treatments were examined 4 weeks after these treatments of subcutaneously transplanted murine syngeneic ED1SQ4 lung cancers. As expected from the presented *in vitro* findings such mitotic cancer cells were detected *in vivo* within xenograft tumors in Figure 6h.

### Chromosome rings and multipolar mitoses are affected by CDK2 but not CDK9 activity

**Figure 7.**
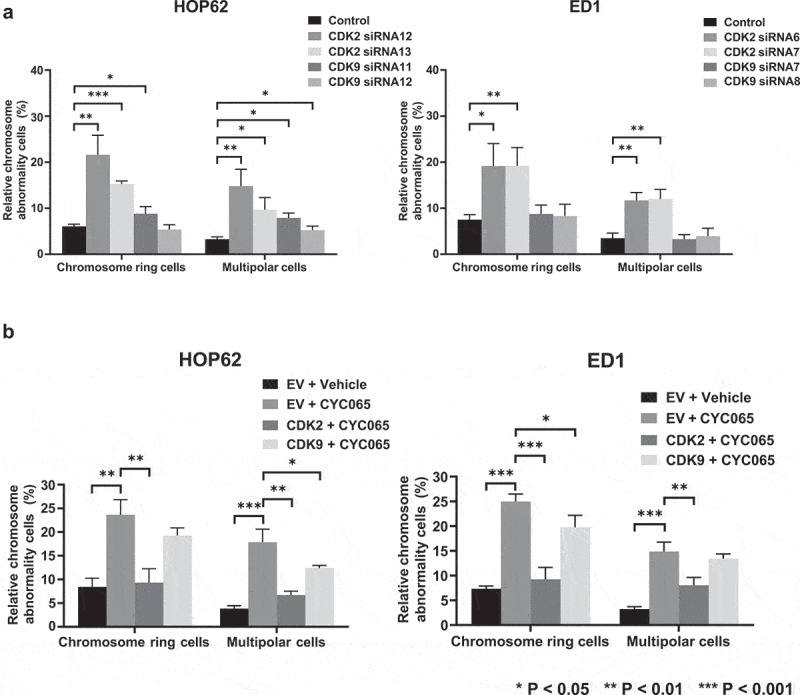
Chromosome rings and multipolar mitoses were prominently observed by CDK2 antagonism (panel a) and not by CDK9 inhibition (panel b) of HOP62 and ED1 lung cancer cells. Knock-down of CDK2 but not CDK9 activities increased chromosome rings or multipolar mitosis as compared to control (inactive) siRNAs transfected independently into HOP62 and ED1 lung cancer cells before immunofluorescent assays were done. Panel b indicated gain of CDK2 but not CDK9 expression antagonized the increase in centriolar abnormalities as well as chromosome rings and multipolar mitoses in HOP62 and ED1 lung cancer cells that were treated with CYC065. Error bars indicated standard deviations with these symbols: **P < .01 and ***P < .001, respectively. In panel b “EV” refers to empty vector.

To determine further the observed pattern of chromosome rings or multipolar mitoses after CDK2 inhibition, CDK2 and CDK9 were individually knocked-down by use of independent transfection of siRNAs in HOP62 and ED1 lung cancer cells (versus control siRNAs). These respective knock-downs were confirmed by immunoblot as well as by real-time PCR assays (Figure S3). Immunofluorescent assays were performed in these CDK2 or CDK9 knocked-down lung cancer cells versus control transfected siRNAs. CDK2 knock-down statistically significantly increased the presence of chromosome rings and multipolar mitoses in the studied cancer cells. In marked contrast, CDK9 knock-down did not exert statistically-significant effects on the appearance of chromosome rings or multipolar mitoses within human HOP62 or murine ED1 lung cancer cells (Figure 7a). Engineered over-expression of CDK2 or CDK9 was independently achieved by transient transfection of these respective expression plasmids within studied lung cancer cells, as confirmed by immunoblot assays (Figure S4).

Immunofluorescent assays in Figure 7 were independently performed in these CDK2, CDK9 or empty vector (EV)-transfected lung cancer cells following treatment with CYC065 (500 nM) or DMSO as a vehicle control. CYC065-treatment statistically-significantly increased the presence of chromosome rings and multipolar mitoses, which were prominently reversed by CDK2, but not by engineered CDK9 over-expression (Figure 7b). Over-expression of CDK2 or CDK9 did not statistically significantly affect chromosome ring or multipolar mitosis formation, as shown in Figure S5. To independently confirm that CDK2 antagonism caused the appearance of lung cancer cells having chromosome rings or multipolar mitoses, CDK2 and CDK2 expression were independently reduced by use of transient transfection of siRNAs and findings were compared to control siRNAs in Figure 7a. These findings revealed that CDK2 knock-down more prominently and statistically significantly promoted chromosome ring and multipolar cancer cell formation than did CDK9 knock-down. Thus, chromosome rings or multipolar mitoses were primarily caused by CDK2 antagonism.

## Discussion

CDK2 inhibition exerts anti-neoplastic effects against diverse aneuploid cancers including lung, colon, and pancreatic cancers. This was reported in our prior work that found the CDK2/9 inhibitor, CYC065, suppressed centrosome clustering and caused anaphase catastrophe.^44–50^ The current study advanced prior work by showing CDK2 inhibition promoted multipolar mitosis and a prolonged state of chromosome rings in cancer cells. This led to failed cytokinesis or cell death (Figures 1 and 2 and data not shown). These outcomes were distinct from previous reported cellular intermediates that occurred before diploid cells enter metaphase.^51^ The studies reported here determined the major cellular fates in cancer cells following CDK2 antagonism.

Taking advantage of the FIB-SEM technique, high-resolution ultrastructure of chromosome rings and multipolar mitoses were displayed in Figure 4a–c. A nuclear morphology abnormality was elucidated by FIB-SEM in Figure 4d. The siRNA knock-down experiments confirmed that chromosome rings and multipolar mitoses were detected after CDK2 inhibition in Figure 7a. Rescue experiments established that engineered CDK2 but not CDK9 over-expression prominently antagonized the formation of chromosome rings and multipolar mitoses caused by CYC065-treatment in Figure 7b. Yet, statistically-significant changes in the presence of chromosome rings or multipolar mitoses were not observed after engineered CDK2 or CDK9 over-expression in murine or human lung cancer cells, as shown in Figure S4.

Chromosome rings were first observed as intermediate structures before bipolar mitotic cells enter metaphase, followed by bipolar spindle separation.^51^ Previously unknown were the fates of cancer cells exhibiting chromosome rings after CDK2 inhibition. We report here that CDK2 inhibition caused a prolonged duration of chromosome rings in the examined cancer cells. This led to the higher frequency of chromosome ring detection in cancer cells versus vehicle-treated controls, as seen in Figure 2a.

Intriguingly, CDK2 inhibition produced cancer cells with chromosome rings. This led to failed cytokinesis and cell death of the treated cancer cells, as in Figure 2b,c. Multipolar mitoses caused by CDK2 inhibition conferred a different fate that led to apoptosis or polynuclear cancer cells along with reduced survival of these progeny. CDK2 inhibition caused diverse abnormalities within mitotic aneuploid cancer cells. One example of this is presented in Figure 4d. The displayed phenotypes could lead to failed nucleokinesis, polynucleated cellular intermediates or another cell cycle without cell division. Future work is warranted to discern the different outcomes within CDK2-inhibited cancer cells. This could provide a functional basis for resistance to a CDK2 inhibitor treatment and lead to formation of residual tumors *in vivo*.

Aneuploidy is a hallmark of cancer.^1^ This can be targeted pharmacologically by inhibition of CDK2 as with CYC065-treatment^48^ and by other drugs, as reviewed.^35^ Our prior work found *in vivo* CDK2 inhibitor treatments of syngeneic mouse lung cancer models (as well as in patient derived lung cancer xenografts and athymic mouse lung cancer xenografts) that statistically-significant reductions of tumor growth followed CDK2-treatment, but residual tumors were detected.^48,49^

The current study examined the effects of CDK2 inhibition in primary cultures of human alveolar epithelial cells. Unlike the outcomes of CDK2-inhibited aneuploid cancer cells, primary cultures of human alveolar epithelial cells were resistant to the growth-inhibitory or proapoptotic effects of this treatment, as displayed in Figure 6f,g. These differences could account for a therapeutic window in which CDK2 inhibitors would preferentially affect malignant cells and tissues while relatively sparing normal cellular components. Unlike in human lung cancer cell lines, CYC065-treatment did not cause an increase in mitotic human alveolar epithelial cells to form chromosome rings or multipolar divisions. Also, there were far fewer mitotic cells in primary cultures of human alveolar epithelial cells than in human lung cancer cell lines. Yet those rare mitotic cells did exhibit centrosome abnormalities in a subset of CYC065-treated cells as shown in Figure 6e (right panel). As expected from the data presented here for cancer cells, murine syngeneic tumors transplanted subcutaneously into recipient mice that were treated with CYC065 or vehicle for 4 weeks exhibited mitotic cancer cells having bipolar, chromosome ring or multipolar morphologies, as in Figure 6h.

The findings presented here are pertinent to the clinical efficacy of CDK2 inhibitors. The abnormal stoichiometry of centrioles described after CDK2 inhibition could confer resistant or persistent tumor cells. This might generate a transient growth-arrested state or the presence of multipolarity that does not elicit cancer cell death but promotes residual tumors. Such tumors could promote a drug-resistant population or seed a recurrent tumor.

One promising strategy to eradicate such residual cancer cells is use of combination therapy. It is intriguing to consider combined use of non-overlapping anti-neoplastics that affect centrosome stoichiometry. One possibility for this to occur is by use of a regimen that combines an optimal CDK2 inhibitor that limits the ability of supernumerary centrosomes to cluster as with a PLK4 antagonist, which we reported to over-duplicate centrosomes in cancer cells.^39^ Such studies are relevant to translational cancer research in that pre-clinical activity could provide a rationale for the design and conduct of a future CDK2 inhibitor trial.

Aneuploidy is a pathognomonic feature of diverse cancers.^1^ CDK2 inhibition that mediated anaphase catastrophe is a broadly engaged mechanism causing a death program in diverse cancers. This includes lymphoma and cancers of lung, colon or pancreatic origins.^48^ This current work provides a basis for hypothesis-driven clinical trials that would explore an optimal CDK2 inhibitor alone or when combined with another centrosome-targeting agent to combat aneuploid cancers. A therapeutic window for this was shown by studies reported here that reveal different outcomes after CDK2 inhibition in primary human alveolar epithelial cells as compared to aneuploid malignant cells. In summary, the findings reported here uncovered a pharmacologically tractable way to counter aneuploid cancers via disruption of centrosomal stoichiometry.

## Methods

### Cell culture

CYC065 was synthesized and obtained from the Division of Cancer Treatment and Diagnosis (DCTD), National Cancer Institute (NCI) (Bethesda, MD). Human lung cancer cell lines (HOP62 and A549), colon cancer (HCT116) and pancreatic (PSN1) cancer cell lines were each purchased and authenticated by ATCC. Murine lung cancer cell lines ED1 and LKR13 cells were independently derived from lung cancers arising from cyclin E overexpressing transgenic mice^54,55^ or Kras^LA1/+^ engineered mice,^56^ respectively, and were authenticated, as described previously.^54–56^ Murine ED1SQ4 lung cancer cells that were used for syngeneic mouse model experiments were derived in our laboratory from ED1 cells.^54^ These cancer cells were each cultured in RPMI 1640 media supplemented with 10% fetal bovine serum (FBS) along with antimycotics and antibiotics at 37°C with 5% CO_2_ in a humidified incubator. Mycoplasma was screened for using the MycoAlert Mycoplasma Detection Kit (Lonza, Basel, Switzerland). All experiments were conducted using cells that tested mycoplasma negative.

### Primary alveolar epithelial cell culture

Human primary alveolar epithelial cells were each purchased and authenticated (Cellbiologics, Chicago, USA). Human alveolar epithelial cells were cultured in Complete Human Epithelial Cell Medium (Cellbiologics, Chicago, USA) supplemented with Insulin-Transferrin-Selenium (ITS), epidermal growth factor (EGF), hydrocortisone, and antibiotic-antimycotic solution in 10% FBS at 37°C with 5% CO_2_ in a humidified incubator. Mycoplasma testing was with the MycoAlert Mycoplasma Detection Kit (Lonza, Basel, Switzerland).

### Plasmids, shRNAs, siRNAs, and transfection procedures

Plasmids HA-CDK2 (#1884, Addgene, Watertown, MA), Flag-CDK9 (#2810, Addgene, Watertown, MA) and empty vector (EX-NEG-M95, Genecopoeia, Rockville, MD) were each purchased. Independent transient transfection experiments were performed with logarithmically growing cancer cells using Lipofectamine 3000 Transfection Reagent (Thermo Fisher Scientific, Waltham, MA). Transfection efficiency was confirmed by immunoblot assays respectively for CDK2 and CDK9, with α-tubulin expression used as a loading control.

Independent transient transfection experiments were accomplished using small interfering RNAs (siRNAs) and the manufacturer’s methods (Dharmacon, Lafayette, CO). Individual siRNAs used to target independently CDK2 or CDK9 as well for inactive control siRNA were synthesized (Dharmacon, Lafayette, CO). Different siRNAs that targeted CDK2 were: human CDK2 siRNA12 (#J-003236-12; 5’-GGACUACCCUCAUGGCCUG-3’); human CDK2 siRNA13 (#J-003236-13; 5’-GCAAAUCUGUCAGUCCAUC-3’); murine CDK2 siRNA6 (#J-040600-06; 5’-CGUUGUUUGUCCAGCACGA-3’) and murine CDK2 siRNA7 (#J-040600-07; (5’-GAUCAUCGGUUCAUGGGAU-3’). Different siRNAs that targeted CDK9 were: human CDK9 siRNA11 (#J-003243-11; 5’-GCAGAUGGCUCGAGAAUAC-3’); human CDK9 siRNA12 (#J-003243-12; 5’-GCCCGUAGGUCAUCUUGGA-3’); murine CDK9 siRNA7 (#J-040602-07; 5’-GCUCGAGAAUACAGAGAGA-3’) and murine CDK9 siRNA8 (#J-040 602-08; 5’- GCAACGAUGUACUGUCUCU −3’). Transfection efficiency was monitored by co-transfecting the siGLO Red Transfection Indicator (Dharmacon, Lafayette, CO) and immunoblot assays of CDK2 or CDK9.

### Immunoblot assays

The indicated cultured cancer cells were lysed with ice-cold Pierce RIPA Lysis and Extraction Buffer (Thermo Fisher Scientific, Waltham, MA) supplemented with Halt Protease and Phosphatase Inhibitor Cocktail (Thermo Fisher Scientific, Waltham, MA). Proteins were resolved by SDS-PAGE before transfer to Trans-Blot Turbo Mini 0.2 µm PVDF Transfer Packs (Bio Rad, Hercules, CA). Membranes were blocked with 5% nonfat milk in Tris-buffered saline (Bio Rad, Hercules, CA) and with 0.1% Tween-20 (Bio Rad, Hercules, CA) (TBS-T) solution for at least 1 hour before overnight incubation at 4°C with a primary antibody diluted in 1% nonfat milk or 1% BSA in TBS-T. This was followed by 10-minute washes x 3 in TBS-T solution and an hour incubation with the desired secondary antibody diluted in 5% nonfat milk. After three additional washes, antibody binding was visualized by Clarity western ECL substrate (Bio Rad, Hercules, CA) and quantified by ImageLab software (Bio Rad, Hercules, CA). Antibodies independently recognized: CDK2 (#ab32147, Abcam, Boston, MA), CDK9 (#ab76320, Abcam, Boston, MA), anti-β-Actin (#4967, Cell Signaling Technology, Beverly, MA), α-tubulin (#3873, Cell Signaling Technology, Beverly, MA), and secondary anti-mouse and anti-rabbit antibodies were also purchased (Bio Rad, Hercules, CA). Immunoblots were stripped using Restore PLUS Western Blot Stripping Buffer (Thermo Fisher Scientific, Waltham, MA).

### Quantitative real-time reverse transcription polymerase chain reaction assays

Quantitative real-time (RT) reverse transcription polymerase chain reaction (PCR) assays were performed as previously described.^57^ The respective primers used were: human CDK2 primer (Hs01548894_m1, Thermo Fisher Scientific, Waltham, MA) and murine CDK2 primer (Mm00443947_m1, Thermo Fisher Scientific, Waltham, MA); human CDK9 primer (Hs00977896_g1, Thermo Fisher Scientific, Waltham, MA) and murine CDK9 primer (Mm01731275_m1, Thermo Fisher Scientific, Waltham, MA); human β-actin primer (Hs01060665_g1, Thermo Fisher Scientific, Waltham, MA) and murine β-actin primer (Mm02619580_g1, Thermo Fisher Scientific, Waltham, MA).

### Live-cell imaging assays

Cells were stained with NucSpot^Ⓡ^ Live Cell Nuclear Stains (#40082, BIOTIUM, Fremont, CA). Washings were with phosphate buffered saline (PBS) before plating in RPMI 1640 media supplemented with 10% FBS with antimycotics and antibiotics. Cells were maintained in the microscope chamber at 37°C and 5% CO_2_. Stained cells were scored for mitotic figures that were bipolar, or those having ring-like chromosomal structures or multipolarity division using a ZEISS Axiocam 506 color microscope and the Live-Cell Imaging system (Carl Zeiss Microscopy LLC, White Plains, NY).

### Immunofluorescent assays

Studied cancer cells were fixed in ice-cold methanol (176845000; Thermo Fisher Scientific, Waltham, MA) and stained with Centrin 1 and γ-tubulin or α-tubulin antibody along with Hoechst staining. Cells were mounted with Pro-Long Gold antifade reagent (P36934; Invitrogen) and scored for chromosome rings and for the presence of multipolar anaphases using a ZEISS Axiocam 506 color microscope with the LSM 900 Airyscan 2 Confocal system (Carl Zeiss Microscopy LLC, White Plains, NY). Primary antibodies recognized: Centrin 1 (12794–1-AP, 1:1000, Proteintech, Rosemont, IL), α-tubulin (T6199; 1:1000, Sigma-Aldrich, St. Louis, MO) or γ-tubulin (T5326; 1:1000, Sigma-Aldrich, St. Louis, MO), respectively. Secondary antibodies were Texas red anti-murine IgG (H+L) (TI-2000; 1:1000, Vector Laboratories, Newark, CA) and goat anti-rabbit IgG (H+L) Cross-Adsorbed Alexa Fluor™ 488 (A11008;1:1000, Thermo Fisher Scientific, Waltham, MA), respectively. Hoechst 33,342 (62249, Thermo Fisher Scientific, 1:10000) stained for DNA. Pro-Long Gold anti-fade reagent preserved immunofluorescence. Each assay was performed in triplicate. Independent triplicate biologic replicate experiments were done.

### Z-stack analyses

Three-dimensional (3D) cell images were acquired for detailed analyses of cancer cells having bipolar, chromosome ring, or multipolar mitoses. This was done after vehicle or CDK2 inhibitor treatments by use of Z-stack image analyses (Range:11 μM, slices: 12 images, Interval: 1 μM) using the Zeiss Axiocam 506 color microscope with the LSM 900 Airyscan 2 Confocal system (Carl Zeiss Microscopy LLC, White Plains, NY).

### Volume electron microscopy: sample preparation

Focused ion beam scanning electron microscopy (CLEM/FIB-SEM) experiments used described methods.^58^ Indicated cancer cell lines were seeded on alphanumerically-coded gridded glass cover slipped wells (MatTek, Ashland, MA). After fixing in 4% paraformaldehyde cells were stained and imaged by fluorescence microscopy. In some experiments live cell imaging was conducted. Brightfield images of the gridded pattern containing the cells were acquired to generate an accurate “target map” of candidate cells for interrogation by FIB-SEM. Cells were rinsed and fixed in Karnovsky’s fixative in 0.1 M sodium cacodylate.

Cells were processed for FIB-SEM using a modified protocol.^58^ Briefly, cells were post-fixed in 2% osmium tetroxide and 1.5% potassium ferricyanide for 1 hour, washed in 0.1 M sodium cacodylate and stained with 1% aqueous uranyl acetate for 1 hour. Samples were washed in water and incubated in Walton’s lead aspartate pH 5.5 for 30 minutes at 60°C. Samples were dehydrated sequentially in 35%, 50%, 70%, 95% and 100% ethanol washes 3 times for 10 minutes each, and in 100% propylene oxide x 3. Specimens were infiltrated in PolyBed resin (Polysciences Inc., Warrington, PA) in a stepwise manner and in 100% resin overnight. After a final 100% resin swap, samples were cured and etched with an alphanumeric pattern. Blocks were cleaned, affixed to an SEM stub with silver paint, dried overnight, and sputter-coated with a layer of carbon before FIB-SEM imaging.

### Image acquisition

Specimens were loaded into the Zeiss Crossbeam 550 FIB-SEM. To locate the cell of interest, an accelerating voltage of 1.5kV or 3kV was used with a beam current of 1.0nA and an SE2 detector. The raised gridded patterns were visible, as were the heavy metal-stained cells. Previously acquired light microscopy images identified target cells of interest. An ATLAS3D (Fibics Inc., Ottawa, Canada) sample preparation workflow was done. A protective platinum pad was overlaid after which tracking and focus lines were milled into the platinum surface and covered by a carbon deposition. A coarse trench and polish mill at 30nA and 3nA, respectively, exposed the cell on the trench face, and an acquisition run was executed. A typical run generated ~ 2000 two dimensional (2D) images.

FIB-SEM reconstructions were analyzed using IMOD (version 4.7) and 3DSlicer (version 4.6) software. The slicer module in IMOD captured 2D images in acquisition and arbitrary planes. ImageJ, iMovie, and Wondershare Filmora generated movies from the respective sections. Segmentation assignments were aided by checking the accuracy of structures in all three planes (xyz) for FIB-SEM image planes. A 3DSlicer was used for visualization and generation of merged FIB-SEM/3D segmentation images.

### Image processing, segmentation, visualization, and quantification

Image stacks were aligned, contrast inverted and binned in the imaging plane using in-house scripts to generate 3D image volumes with isotropic voxels (typically 15 nm to a side). Where DNA and centrioles needed to be segmented and analyzed the deep learning (DL) module in Arivis vision4D was used. After running DL model inference, the output segmentation was manually cleaned and smoothed in all three planes. Centrioles were manually segmented using Slicer3D or Amira, using contrast thresholding and manual polishing. Volume renderings of cellular features and movies were made using Amira. Centriole measurements were with Slicer3D. An average of six measurements were taken at different points along the long axis for length estimates, or radially at proximal and distal ends for width measurements of examined centrioles or centrosomes.

### Mouse model experiments

Marked anti-tumorigenic effects following oral gavage of CYC065-treatment as compared to vehicle controls (Phosal 60%, PEG400 30% and 10% EtOH) were reported using murine ED1SQ4 syngeneic xenografts in 6–8-week-old male immunocompetent 129S2/SVPasCrl mice (Charles River Laboratories).^57^ In this study those anti-tumorigenic effects were analyzed at 4 weeks of CYC065 versus control treatment by examining formalin-fixed and paraffin-embedded tumors harvested from mice. These tumors were immunostained with phospho-histone H3 (Ser10) antibody (9701, Cell Signaling Technology; 1:200) and counterstained with hematoxylin. This facilitated the scoring of mitotic figures for the presence of bipolar cancer cells as well as those cancer cells exhibiting chromosome rings or multipolarity. These experiments were conducted following a study protocol (#100161) approved by the National Cancer Institute (NCI) Animal Care and Use Committee (ACUC).^57^

### Statistical analysis

Statistical analyses were with SPSS Statistics software (version 23, SPSS) and GraphPad Prism software (version 8, GraphPad Software). Data were presented as mean with Standard Deviation (SD) displayed. Two-tailed Student t tests compared differences between study groups with a *P* value < 0.5 deemed significant. Results of independent experiments were pooled to assess statistical significance. Experiments were performed in triplicate with at least three independent replicate experiments.

## Supplementary Material

Supplemental Material

## Data Availability

The datasets used as well as analyzed for this study will be available from the corresponding author upon reasonable request.
