# Supplementary material for: CDK2 inhibition disorders centrosome stoichiometry and alters cellular outcomes in aneuploid cancer cells
Source: Cancer Biol Ther. 2023 Nov 30;24(1):2279241. doi: 10.1080/15384047.2023.2279241 (PMC10766391; doi:10.1080/15384047.2023.2279241)

##
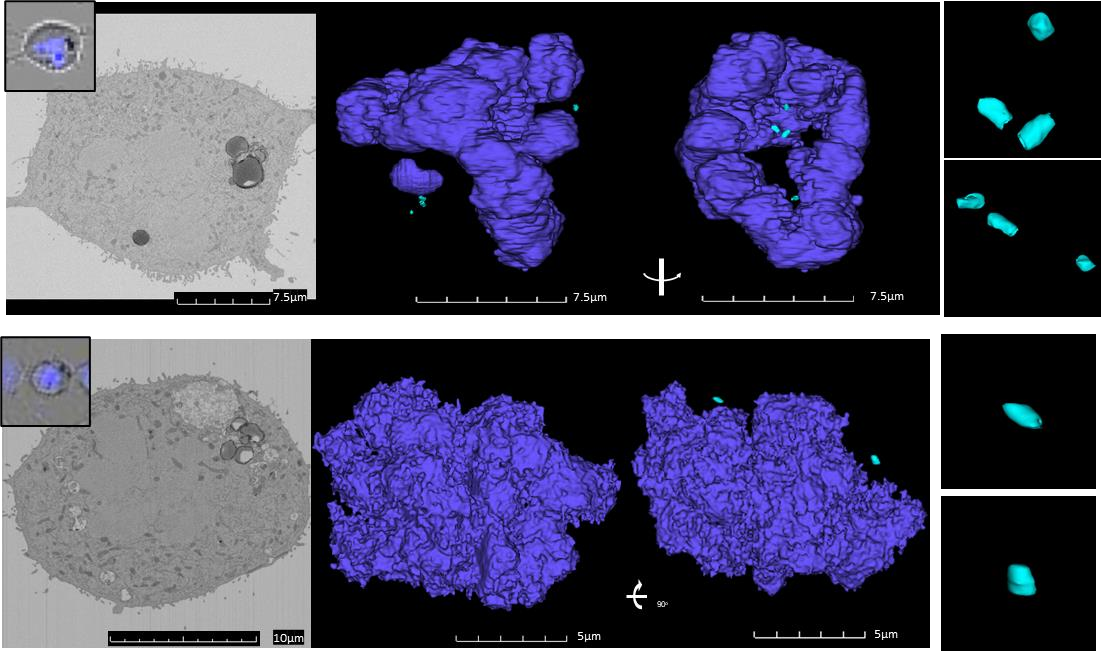
a

**b**

**Supplementary Figure 1**: Correlation between nuclear morphology and centriolar localization in CYC065-treated HOP62 lung cancer cells as compared to vehicle controls showing: (**a**) paired and (**b**) unpaired arrangements of centrioles, respectively.

|  | **Chromosome ring cells** | | | | **Multipolar cells** | | | **Bipolar cells** | | |
| --- | --- | --- | --- | --- | --- | --- | --- | --- | --- | --- |
|  | **Abnormal number of centrioles** | | **Normal centriole number and percent of centrosomes** | **Total abnormal percent of centrioles** | **Abnormal number of centrioles** | **Normal number of centrioles** | **Abnormal percent of centrioles** | **Abnormal number of centrioles** | **Normal number of centrioles** | **Abnormal percent of centrioles** |
|  | **Single centrosome** | **Dual centrosomes** |  |  |  |  |  |  |  |  |
| **HOP62**  **Vehicle** | **0 (0%)** | **12 (16.0%)** | **63 (84%)** | **16.0%** | **9** | **40** | **18.4%** | **39** | **344** | **10.2%** |
| **HOP62 CYC065 (500nM)** | **7 (10.1%)** | **43 (62.3%)** | **19 (27.5%)** | **72.5%** | **50** | **7** | **87.7%** | **82** | **263** | **23.8%** |
| **ED1**  **vehicle** | **6 (6.8%)** | **6 (6.8%)** | **76 (86.4%)** | **13.6%** | **4** | **24** | **14.3%** | **18** | **303** | **5.6%** |
| **ED1 CYC065 (500nM)** | **29 (30.5%)** | **42 (44.2%)** | **24 (25.3%)** | **74.7%** | **24** | **8** | **80%** | **38** | **312** | **10.9%** |

**Supplementary Figure 2**: CYC065-treatment caused abnormal centriolar stoichiometry in HOP62 and ED1 lung cancer cells, respectively. Displayed here is the quantification of each of these outcomes in HOP62 and ED1 lung cancer cells treated with vehicle or CYC065 (500 nM).

# HOP62 ED1

**Con**

**siRNA**

**siRNA**

**Con**

**siRNA siRNA**

**KDa 37**


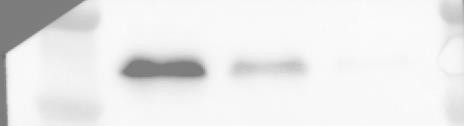

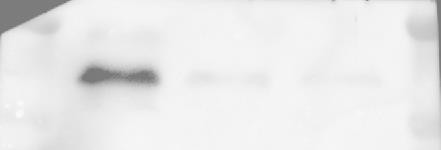


**siRNA 12 13**

**KDa 37**

**siRNA 6 7**

# CDK2 CDK2

**25 25**

**75 75**


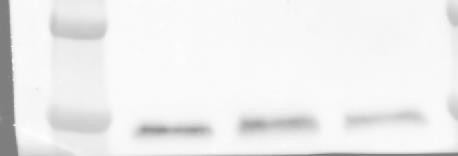

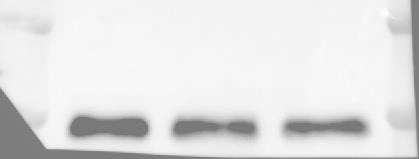


## α-Tubulin α-Tubulin

**50 50**

**100 100**

**Relative CDK2 expression (%)**

**Relative CDK2 expression (%)**

**80 80**

**60 60**

**40 40**

**20**

**0**

**CDK2 CON CDK2 siRNA12CDK2 siRNA13**

# HOP62

**20**

**0**

**CDK2 CON CDK2 siRNA6 CDK2 siRNA7**

# ED1

**Con**

**siRNA siRNA**

**Con**

**siRNA siRNA**

# CDK9

**KDa 50**

**37**


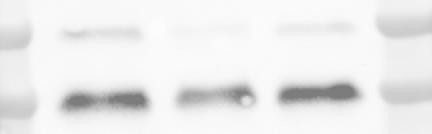


**75**


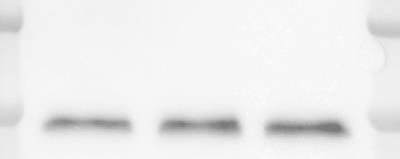


**siRNA 11 12**

# CDK9

**KDa**

**50**

**37**

**75**


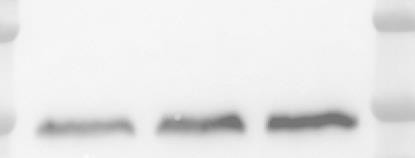


**siRNA 7 8**


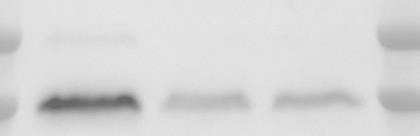


## α-Tubulin

**50**

## α-Tubulin

**50**

**100**

**Relative CDK9 expression (%)**

**80**

**60**

**40**

**20**

**0**

## c

**CDK9 CON CDK9 siRNA11 CDK9 siRNA12**

**100**

**80**

**Relative CDK9 expression (%)**

**60**

**40**

**20**

**0**

**CDK9 CON CDK9 siRNA7 CDK9 siRNA8**

**100**

**CDK2 Relative mRNA level (%)**

**80**

**60**

**40**

**20**

**0**

**HOP62**

✱


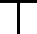

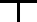


✱✱✱

**Control siRNA12 siRNA13**

**100**

**80**

**CDK2 Relative mRNA level (%)**

**60**

**40**

**20**

**0**

**ED1**

✱✱✱

✱✱✱

**Control siRNA6 siRNA7**

**100**

**80**

**CDK9 Relative mRNA level (%)**

**60**

**40**

**20**

**0**

**HOP62**

✱✱✱


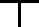


✱✱✱

**Control siRNA11 siRNA12**

**100**

**80**

**CDK9 Relative mRNA level (%)**

**60**

**40**

**20**

**0**

**ED1**

✱✱✱

✱✱✱

**Control siRNA7 siRNA8**

**Supplementary Figure 3**: CDK2 and CDK9 knock-downs were respectively achieved by use of individual siRNAs that repress these species in HOP62 and ED1 lung cancer cell lines. Findings were compared to control (Con) siRNAs. (**a-b**) Immunoblot assays for CDK2 and CDK9 expression profiles were each done to validate these knock-downs. In panel (**a**) one membrane was cut and probed for CDK2 and α-tubulin protein expression separately. In panel (**b**) the CDK9 immunoblot was imaged first, followed by stripping and probing for α-tubulin protein expression. All results were quantified by Imagine Lab software. (**c**) RT-quantitative PCR assays for CDK2 and CDK9 expression profiles were each done to validate these knock-downs. The symbols indicate * P < 0.05 and *** P < 0.001.

**a HOP62**

**ED1**

**HOP62**

**ED1**

**KDa**

**250**

**150**

**100**

**75**

**50**

**37**

**25**

**20**

**10**

## b

**c**

**EV CDK2 EV CDK2**


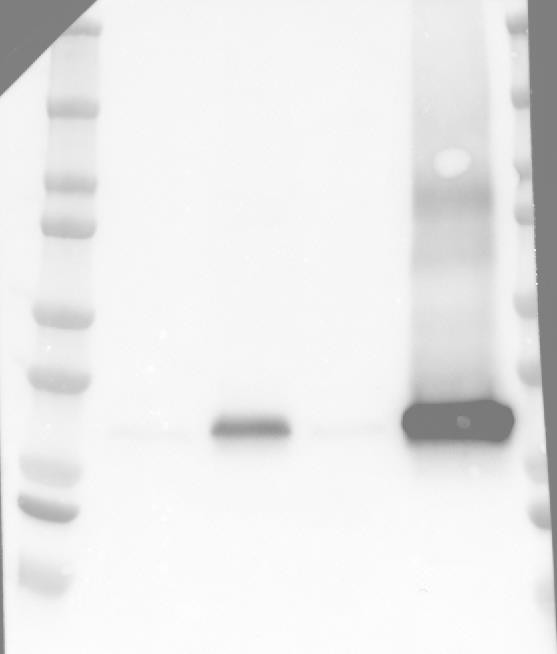


**HOP62 CDK2 overexpression**

**5**

**Relative CDK2 expression**

**4**

**3**

**2**

**1**

**0**

**EV CDK2**

**CDK2**

**KDa**

**250**

**150**

**100**

**75**

**50**

**37**

**25**

**20**

**10**

**EV CDK2 EV CDK2**


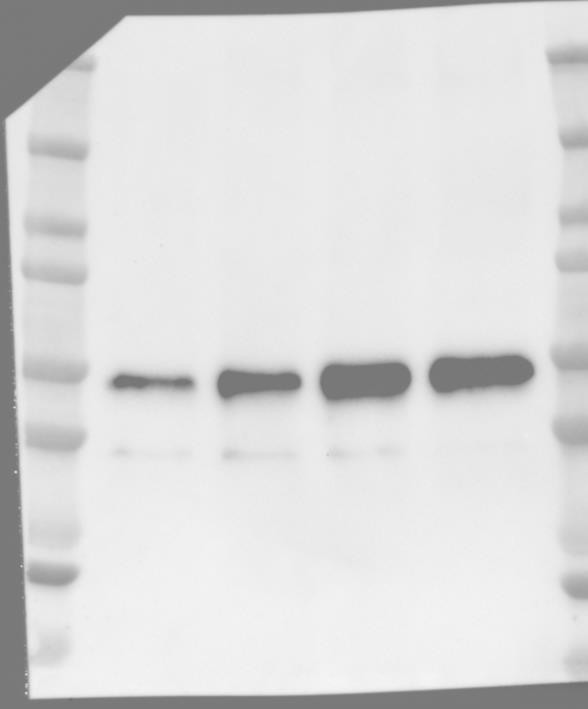


**ED1 CDK2 overexpression**

**20**

**Relative CDK2 expression**

**15**

**10**

**5**

**0**

**EV CDK2**

**α-Tubulin**

**HOP62**

**ED1**

**HOP62**

**ED1**

**KDa**

## d

**3**

**Relative CDK9 expression**

**2**

**EV CDK9 EV CDK9**


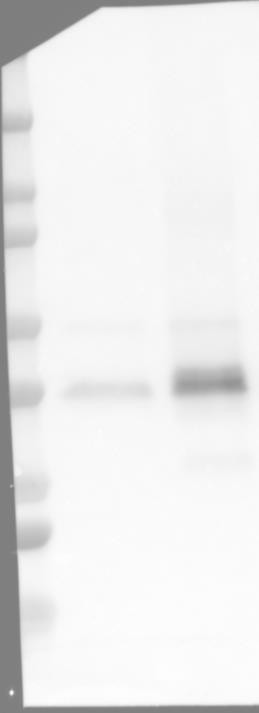


**250**

**150**

**100**

**75**

**50**

**37**

**25**

**20**

**10**


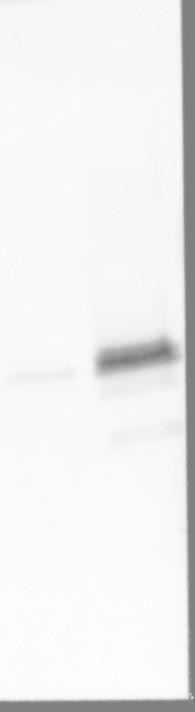


**HOP62 CDK9 overexpression**

**CDK9**

**KDa**

**250**

**150**

**100**

**75**

**50**

**37**

**25**

**20**

**10**

**10**

**Relative CDK9 expression**

**8**

**6**

**EV CDK9 EV CDK9**


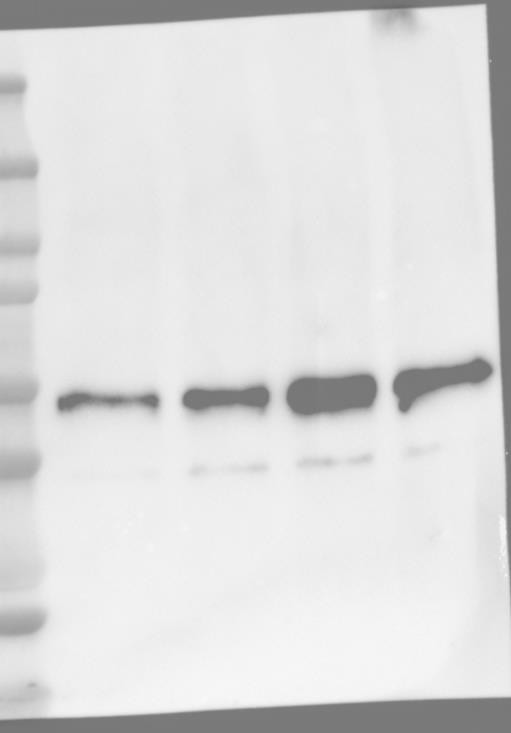


**ED1 CDK9 overexpression**

**α-Tubulin**

**1**

**0**

**EV CDK9**

**4**

**2**

**0**

**EV CDK9**

**Supplementary Figure 4**: Independent gain of CDK2 or CDK9 expression by transient transfection of CDK2 or CDK9 expression plasmids was independently achieved in HOP62 and ED1 lung cancer cell transfectants. (**a-b**) Immunoblot assays were done and quantified to validate over-expression of CDK2. (**c-d**) Immunoblot assays were done and quantified to validate over-expression of CDK9. For each immunoblot, the same amount of lysate proteins from each respective transfectant was loaded for immunoblot determination of CDK2, CDK9 and α-tubulin expression profiles. All results were quantified by Imagine Lab software. In this figure “EV” refers to empty vector.

## a

### HOP62

**EV+Vehicle**

**20 CDK2+Vehicle**

**CDK9+Vehicle**

**10**

**Relative chromosome abnormality cells (%)**

**0**

**Chromosome ring cells**

## b

**20**

### ED1

**Multipolar cells**

**EV+Vehicle CDK2+Vehicle CDK9+Vehicle**

**10**

**Relative chromosome abnormality cells (%)**

**0**

**Chromosome**

1. **ring cells**

**Multipolar cells**

**HOP62**

**ED1**

**HOP62**

**ED1**

**HOP62**

**ED1**

**HOP62**

**ED1**

**KDa**

**250**

**150**

**100**

**75**

**50**

**37**

**25**

**20**

**10**

**EV CDK2 EV CDK2**

**CDK2**

**KDa**

**250**

**150**

**100**

**75**

**50**

**37**

**25**

**20**

**10**

**EV CDK2 EV CDK2**


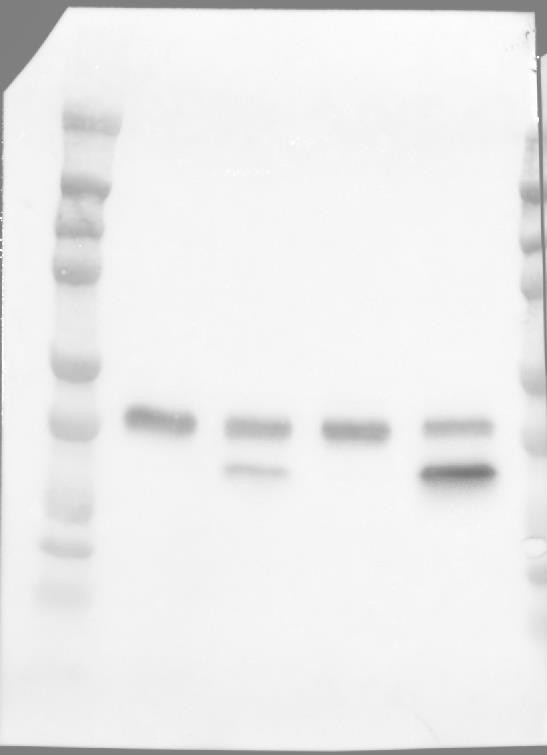


**β-Actin**

**KDa**

**250**

**150**

**100**

**75**

**50**

**37**

**25**

**20**

**10**

**EV CDK9 EV CDK9**

**KDa**


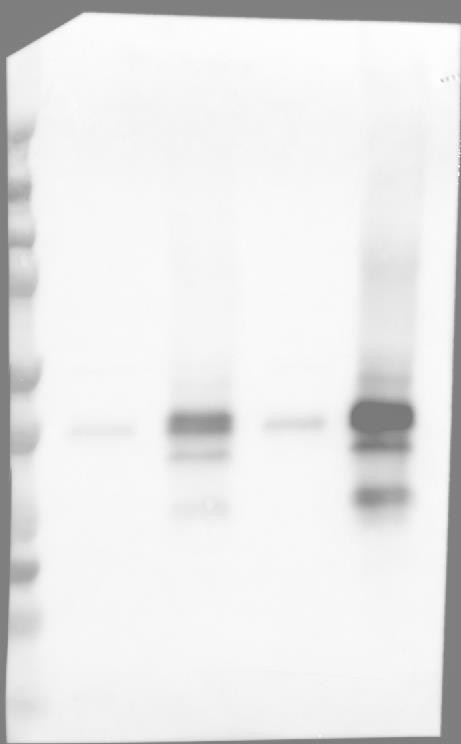

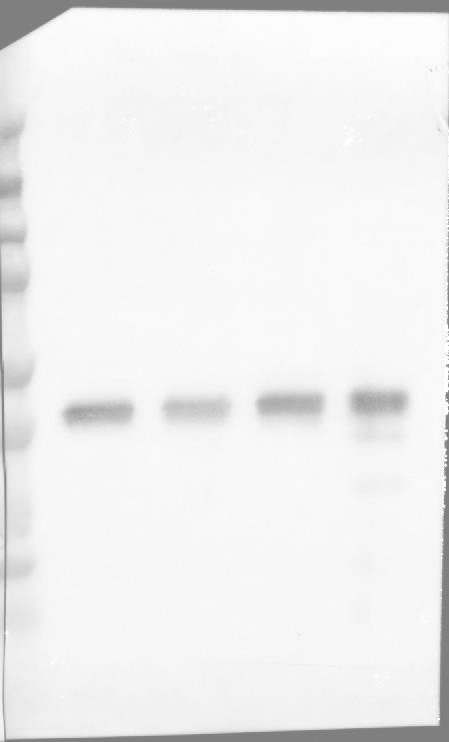


**EV CDK9 EV CDK9**

**250**

**150**

**100**

**75**

**50**

**CDK9**

**37**

**25**

**20**

**10**

**β-Actin**

**Supplementary Figure 5**: CDK2 or CDK9 overexpression did not induce chromosome ring and multipolar mitoses in HOP62 and ED1 lung cancer cell lines. Immunofluorescence assays were done and scored for the proportion of chromosome rings and multipolar mitoses in (**a**) HOP62 and (**b**) ED1 cells that express an empty vector (EV), CDK2 expression vector, or CDK9 expression vector along with vehicle (DMSO) treatment. Error bars indicated standard deviations. (**c**) Immunoblot assays were done and quantified to validate over-expression of CDK2 and CDK9. For each immunoblot, the same amount of lysate proteins from each respective transfectant was loaded for immunoblot determination of CDK2, CDK9 and β-Actin expression profiles.


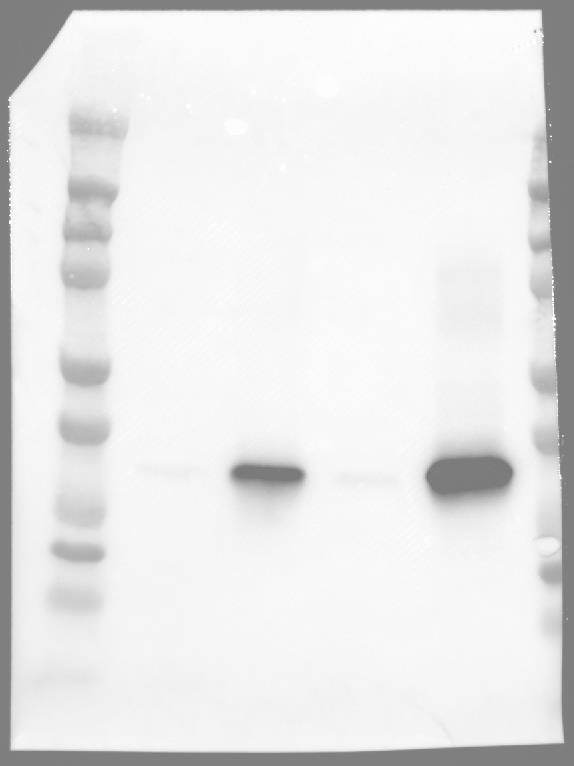

Supplement: Supplemental Material [file KCBT_A_2279241_SM5481.docx]
